# Neuronal Imaging at 8‐Bit Depth to Combine High Spatial and High Temporal Resolution With Acquisition Rates Up To 40 kHz

**DOI:** 10.1002/jbio.202400513

**Published:** 2025-01-10

**Authors:** Fatima Abbas, Ömer Yusuf İpek, Philippe Moreau, Marco Canepari

**Affiliations:** ^1^ Univ. Grenoble Alpes, CNRS, LIPhy Grenoble France; ^2^ Laboratories of Excellence Ion Channel Science and Therapeutics Valbonne France; ^3^ Institute of Health Sciences/Physiology, Erciyes University Kayseri Turkey; ^4^ Department of Physiology, Faculty of Medicine Kirşehir Ahi Evran University Kirşehir Turkey; ^5^ Institut National de la Santé et Recherche Médicale Paris France

**Keywords:** brain slices, calcium imaging, neurons, voltage imaging

## Abstract

A challenge in neuroimaging is acquiring frame sequences at high temporal resolution from the largest possible number of pixels. Measuring 1%–10% fluorescence changes normally requires 12‐bit or higher bit depth, constraining the frame size allowing imaging in the kHz range. We resolved Ca^2+^ or membrane potential signals from cell populations or single neurons in brain slices by acquiring fluorescence at 8‐bit depth and by binning pixels offline, achieving unprecedented frame sizes at kHz rates. In hippocampal slices stained with the Ca^2+^ indicator Fluo‐4 AM, we resolved transients at 2 kHz from large frames. Along the apical dendrite of a layer‐5 pyramidal neuron, we measured Ca^2+^ signals associated with a back‐propagating action potential at 10 kHz. Finally, in the axon initial segment of the same cell type, we recorded an action potential at 40 kHz by voltage‐sensitive dye imaging. This approach unlocks the potential for a range of imaging measurements.

## Graphical abstract

We demonstrate that fluorescence transients from calcium or from voltage‐sensitive dyes, associated with neuronal signals, can be resolved by acquiring frames at 8‐bit digital depth and by binning pixels offline. With this approach, we could attain exceptional frame sizes, in terms of number of pixels, at very high temporal resolution.

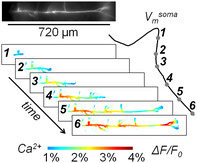

## 1 Introduction

Optical recordings of membrane potential (*V*_m_) and intracellular ion transients are commonly used to investigate multisite neuronal activity [1, 2]. These recordings are measurements of fluorescence from a dye, which can be a genetically encoded protein [3] or an organic indicator [4]. When using wide‐field illumination to measure signals from multiple neurons, or multiple sites of a single neuron, the appropriate recording device should be an array of many detectors with high quantum efficiency (QE), as well as high spatial and temporal resolution. In particular, when measuring an action potential (AP) with a voltage‐sensitive dye (VSD), or an ionic transient associated with an AP, acquisitions should be performed in the kHz range. For a long time, this was achievable using photodiode arrays with a limited number of detectors [5, 6], until advancements in digital camera technology [7, 8] made it possible to achieve fast imaging at higher spatial resolution. The first fast high QE cameras were based on charge‐coupled device (CCD) technology, which uses an efficient strategy for signal readout. As a reference, a widely exploited CCD camera composed of 80 × 80 pixels was used to measure *V*_m_ fluorescence [9, 10] or Na^+^ and Ca^2+^ fluorescence [11] at full‐frame resolution at 2 kHz. Since in CCD cameras photoelectrons in readout register are shifted horizontally onto the output node before being amplified and converted to a digital signal, the acquisition rate can be increased by reducing the vertical dimension of the frame or by binning the frame. Thus, using the same camera mentioned above, it was possible to acquire 80 × 12 pixels frames at 10 kHz [12], or 26 × 26 binned‐pixel frames at 5 kHz [13, 14]. Today, complementary metal‐oxide semiconductor (CMOS) technology offers superior performance in achieving a combination of high spatial and temporal resolution [15]. Using a CMOS camera, one‐dimensional recordings at > 100 kHz could be achieved [16]. Like CCDs, CMOS cameras can increase acquisition speed by reducing the vertical frame dimension. Thus, using an advanced CMOS camera, we were able to measure *V*_m_, Na^+^ and Ca^2+^ fluorescence at 10 kHz with a frame size of 128 × 30 pixels [17, 18], significantly increasing the frame size that could be achieved at the same rate using a CCD camera. A critical step in the readout process, which limits the maximal acquisition rate of a camera at a given frame size, is the bit depth of the analog‐to‐digital (A/D) converter. Since a typical fractional fluorescence change (Δ*F*/*F*_0_) associated with ionic or *V*_m_ transients ranges from 0.1% to 10%, and the background fluorescence in a preparation is often not uniform, A/D converters with a minimum of 12‐bit depth have been selected for these applications to ensure sufficient sensitivity and precision. The frame size that can be achieved at high bit depth (see above), compels either reducing the acquired field size to maintain the area covered by a pixel at ~1 μm or below, or degrading the image with pixels covering several microns to record from larger fields. The camera speed, however, can dramatically increase if the analog signal is converted to an 8‐bit digital signal and, theoretically, the effective digital depth of the signal can be increased by summing the digital values of adjacent pixels allowing signal detection beyond the limitation of digital noise. Here, we report how a fast CMOS camera with more than 10 million pixels can resolve various types of fast neuronal signals, corresponding to Δ*F*/*F*_0_ transients of 1%–10%, by converting low light levels to 8‐bit digital signals. To achieve this, we used a simple post‐acquisition processing procedure that we name *N* × *N offline binning*. This method assigns to each pixel the summation of digital signals over the *N* × *N* rectangle surrounding it. Since summation of 2 × 2 8‐bit value gives a 10‐bit value, and summation of 4 × 4 8‐bit value gives a 12‐bit value, offline binning increases both the light level of the pixel and its effective digital depth. The process is equivalent to averaging fluorescence over pixels belonging to a *region of interest* (ROI), which is routinely done when imaging neuronal signals with cameras to increase the signal‐to‐noise ratio (SNR) with respect to the intrinsic photon noise. The improved acquisition speed at 8‐bit depth allowed recording frames with previously unattainable numbers of pixels at a given speed, or to resolve signals at exceptional time resolution, as demonstrated in three distinct examples.

## 2 Materials and Methods

### 2.1 Ethical Approval

Experiments in brain slices were performed at the Laboratory of Interdisciplinary Physics in Grenoble in accordance with European Directives 2010/63/UE on the care, welfare and treatment of animals. Procedures were reviewed by the ethics committee affiliated to the animal facility of the university (E3842110001). Mice (C57BL/6j) were kept in the animal house and fed *ad libitum* until euthanasia. They were anesthetised by isoflurane inhalation and the entire brain was removed after decapitation.

### 2.2 Slice Preparation and Solutions

Transversal (horizontal) hippocampal slices were prepared from 5 postnatal weeks old mice using a Leica VT1200 (Leica, Wetzlar, Germany) as previously described [19, 20], in this case with a thickness of 350 μm. Neocortical slices (350 μm thick) were prepared from 4 postnatal weeks old mice following established protocols [17, 18, 21]. The extracellular solution contained (in mM): 125 NaCl, 26 NaHCO_3_, 20 glucose, 3 KCl, 1 NaH_2_PO_4_ and 2 CaCl_2_ bubbled with 95% O_2_ and 5% CO_2_. After slicing, all slices were pre‐incubated for 45 min at 37°C and then maintained at room temperature. Slices were finally transferred to the recording chamber under an Olympus BX51 microscope where they were perfused with extracellular solution at 32°C–34°C. In one experiment used in this report, the perfusing solution contained 10 μM of the GABA_A_ receptors antagonist bicuculline (purchased from HelloBio, Dunshaughlin, Republic of Ireland). In the two experiments involving patch clamp recordings, the intracellular solution contained (in mM): 125 KMeSO_4_, 5 KCl, 8 MgSO_4_, 5 Na_2_‐ATP, 0.3 Tris‐GTP, 12 Tris‐Phosphocreatine, 20 HEPES, adjusted to pH 7.35 with KOH.

### 2.3 Ca^2+^ and VSD Loading

Before being transferred to the recording chamber, hippocampal slices were stained with Fluo‐4 AM ester (from Biotium, Fremont, CA) using a procedure similar to a previous one with Fluo‐3 AM ester [22, 23]. Briefly, 50 μg vials of Fluo‐4 AM ester were pre‐dissolved at 5 mM in DMSO and slices were incubated at room temperature in a solution containing 2–3 μM of the dye for 30–45 min. In the experiment using the Ca^2+^ indicator Oregon Green BAPTA‐5N (OG5N, Thermo Fisher Scientific), the dye was dissolved in the intracellular solution at 2 mM concentration and the cell was loaded through a patch pipette [21]. In the experiment using the VSD D‐2‐ANEPEQ (JPW1114, Thermo Fisher Scientific), the dye was dissolved in the intracellular solution at 0.2 mg/mL concentration and the cell was loaded through a patch pipette using a previously described procedure [19].

### 2.4 Imaging in Brain Slices and Electrophysiology

**FIGURE 1:**
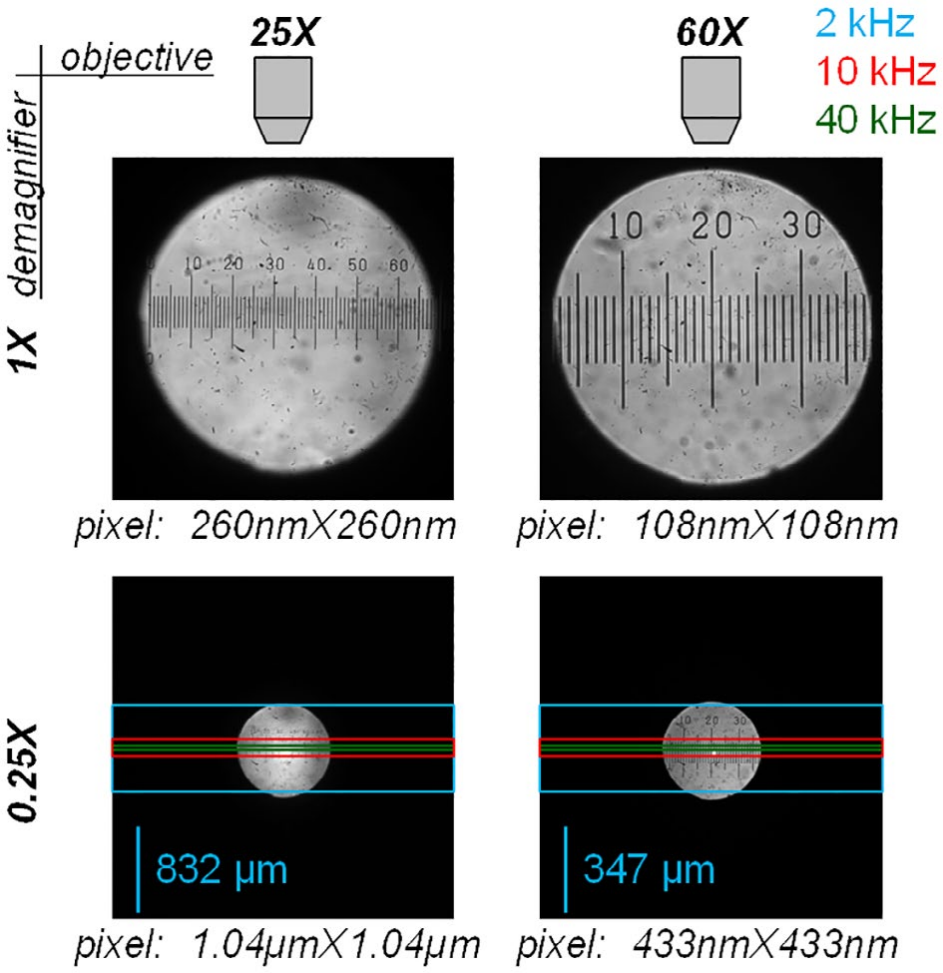
Fields of view of a 25× objective (left) and of a 60× objective (right) captured by the camera with 1× demagnification (top) and 0.25× demagnification (bottom). The pixel sizes in all configurations are reported. The light blue, red and green rectangles represent the theoretical maximal sizes of the frames that can be acquired at 2, 10 and 40 kHz according to the camera datasheet.

The microscope was equipped either with a 25×/1.05 NA objective (model XLPLN25XWMP2) or with a 60× Olympus water immersion objective (NA = 1) and images were acquired with a Kinetix (Teledyne Photometrics, Tucson, AZ) CMOS camera (3200 × 3200 pixels). Without magnification (1 × demagnifier, Figure 1, top), the camera is capable of visualising the entire field of view, which is ~720 μm diameter for the 25 × objective and ~ 320 μm diameter for the 60 × objective. However, to image larger portions of the field when acquiring frames at 8‐bit depth, we used a 0.25 × demagnifier, obtaining a pixel size of 1.04 μm with the 25 × objective and 433 nm when using the 60 × objective (Figure 1, bottom). The light blue, red and green rectangles in Figure 1 (bottom), represent the theoretical maximal sizes of the frames that can be acquired at 2 kHz (3200 × 800 pixels), 10 kHz (3200 × 160 pixels) and 40 kHz (3200 × 40), according to the camera datasheet. In practice, in order to achieve adequate exposure time to collect enough photons, the frame sizes used in the proof‐of‐principle experiments were smaller (see Results). In Ca^2+^ imaging recordings, Fluo‐4 or OG5N were excited by the 470 nm line of an OPTOLED (Cairn Research, Faversham, UK) and the emitted fluorescence was band‐pass filtered at 530 ± 21 nm before being acquired. In the *V*_m_ imaging experiment, JPW1114 fluorescence was excited using the 528 nm line of an LDI‐7 laser (89 North, Williston, VT) and the emitted fluorescence was long‐pass filtered at > 610 nm before being acquired. In the experiments involving patch clamp recordings, we used a Multiclamp 700A (Molecular Devices, Sannyvale, CA). A Somatic AP was elicited in current clamp mode by a short current pulse of 1.5–2.5 nA through the patch pipette and electrical signals were acquired at 40 kHz.

### 2.5 Data Recording and Analysis

Images were acquired with the open source software Micro‐Manager. The exact inter‐frame interval was set by adjusting the exposure time parameter in the software and measured by using an LED with a rise time < 10 μs. To further ensure accurate timing, the frame rate was verified by capturing the “exposure output” channel from the camera using a National Instruments USB‐6343 interface. The same device was used to acquire the *V*_m_ signal from the Multiclamp 700A that was corrected for the junction potential (−11 mV) while the AP bridge was corrected offline by using the recorded injected current. Data were analysed in MATLAB. Frame sequences, either from single trials or from averages of 3–9 trials with identical responses, were corrected for photo‐bleaching by subtracting the frame sequence from a trial without stimulation. Signals were expressed as Δ*F*/*F*_0_. When averaged within defined regions, a Savitzky–Golay smoothing filter [24] was applied to the trial without stimulation to minimise the additional noise introduced by bleaching correction. For the creation of colour‐scaled frames and videos, a mask was applied to remove dark pixels and Δ*F*/*F*_0_ frames were illustrated using a colour scale. In Video S1 and Video S2 (available in the Supporting Information), each frame is the average of four original frames and movies were displayed at 10 frames/s without the colour scale. Finally, this is a technical report where only proof‐of‐principle results are presented. Therefore, no statistical analysis was performed.

## 3 Results

### 3.1 Principles of 8‐Bit Imaging and Offline Binning

**FIGURE 2:**
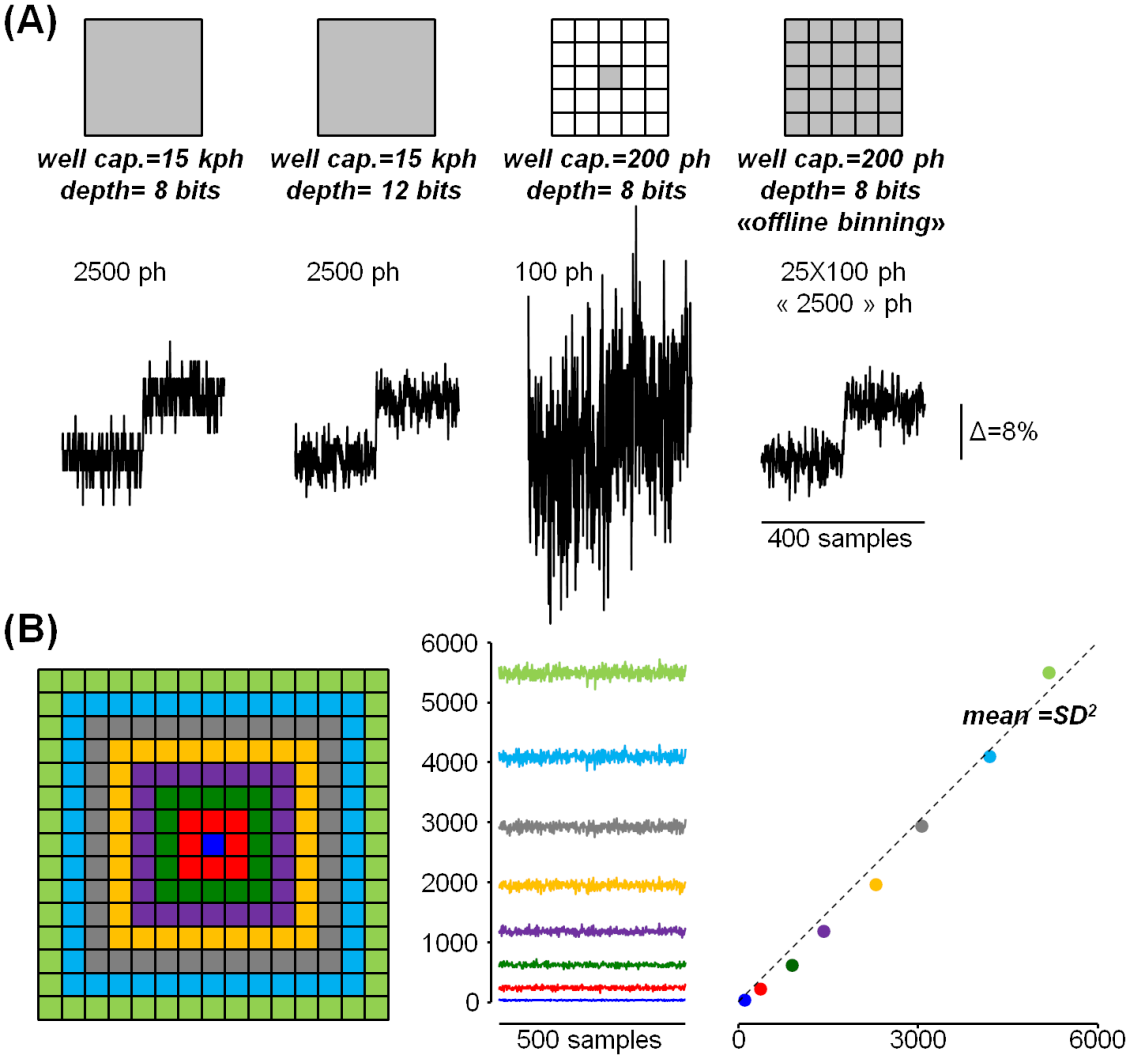
Principles of 8‐bit imaging and offline binning. (A) Left, randomly generated 400 numbers following a Gaussian distribution with mean stepping from 2500 to 2700 and SD equal to 50 simulating a 8% Δ*F*/*F*_0_ signal from *F*_0_ = 2500 photons recorded by a pixel; the signal is converted either to 8‐bit or to 12‐bit. Right, randomly generated numbers following a Gaussian distribution with mean stepping from 100 to 108 and SD equal to 10 simulating a 8% Δ*F*/*F*_0_ signal from F_0_ = 100 photons converted to 8‐bit; traces are either from a single pixel or the summations of number series of digital numbers from 25 pixels, representing 5 × 5 offline binning. (B) Left, digital numbers corresponding to uniform fluorescence recorded with the CMOS camera in 8‐bit mode, from a single pixel (blue trace) and by applying 3 × 3‐, 5 × 5‐, 7 × 7‐, 9 × 9, 11 × 11, 13 × 13‐ and 15 × 15 offline binning. Right, plot of SD^2^ against mean of the digital numbers from the traces on the left; the dotted line superimposed is the linear curve with coefficient 1, indicating that a digital unit corresponds to ~1 photoelectron.

In camera imaging, the physical *well capacity* of a pixel sets the maximum number of photoelectrons that can be measured. However, since the analog signal is converted to a digital signal, the “effective well capacity” (EWC) is eventually the key parameter as it corresponds to the number of photoelectrons equivalent to the maximal digital number, which is determined by signal amplification before conversion. Thus, if *β* is the bit depth of the converted signal, the number of photons that can be discriminated is always > EWC/2^β^. In practice, this number must be considered against the photon noise, which is given by the square root (√) of the mean number of detected photoelectrons (γ). In the computer simulation shown in Figure 2A (left), we randomly generated 400 numbers using a Gaussian distribution with γ equal to 2500 and SD equal to 50 (√γ). We assumed EWC = 15 000 and we converted the simulation values either to 8‐bit (EWC/2^β^ = 15 000/256 ~ 59) or to 12‐bit (EWC/2^β^ = 15 000/4096 ~ 4). As shown in Figure 2*A* (left), a change of 8% from γ (200) can be detected both at 8‐bit and at 12‐bit depths, but while at 12‐bit depth the Gaussian noise dominates, the digital noise becomes dominant at 8‐bit depth. We then performed 25 computer simulations randomly generating numbers from a Gaussian distribution with γ equal to 100 and SD equal to 10 (√100). In this case, we assumed that the values of the simulations were collected from 25 pixels with EWC = 200 and *β* = 8. As shown in Figure 2A (right), a change of 8% from γ (100·0.08 = 8) cannot be discriminated by a single pixel because this change is below the Gaussian noise (√100 = 10). But when the values are summed over the 25 pixels, that is, when we applied 5 × 5‐pixels offline binning, the 8% change corresponding (again) to 200 is above the Gaussian noise (√2500 = 50). In this case, however, the digital discrimination limit is EWC/2^β^ = 200/256 (< 1) and it is negligible with respect to the Gaussian noise (Figure 2A, right). The datasheet of the camera that we used in this study states that EWC = 200 when sampling at 8‐bit depth, corresponding to the scenario of the computer simulations reported in Figure 2*A* (right). To confirm this information, we collected uniform light intensity from a fluorescent coverslip, tuning the excitation power to obtain an average digital value of ~100 (Figure 2B, left). We calculated the mean and SD from 500 samples starting from one pixel and continuing by applying 3 × 3‐, 5 × 5‐, 7 × 7‐, 9 × 9, 11 × 11, 13 × 13‐ and 15 × 15‐pixels offline binning. As shown in Figure 2B (right), the plot of the mean versus the square of the SD appears linear with coefficient ~ 1, indicating that each digital value corresponds to ~1 photoelectron. In the proof‐of‐principle examples of the following sections, we will demonstrate the ability of 8‐bit recordings combined with offline binning to resolve Ca^2+^ and *V*_m_ fluorescence transients with unprecedented spatial and temporal resolution.

### 3.2 Imaging Ca^2+^ Transients at 2 kHz in Hippocampal Slices Stained With Fluo‐4 AM

**FIGURE 3:**
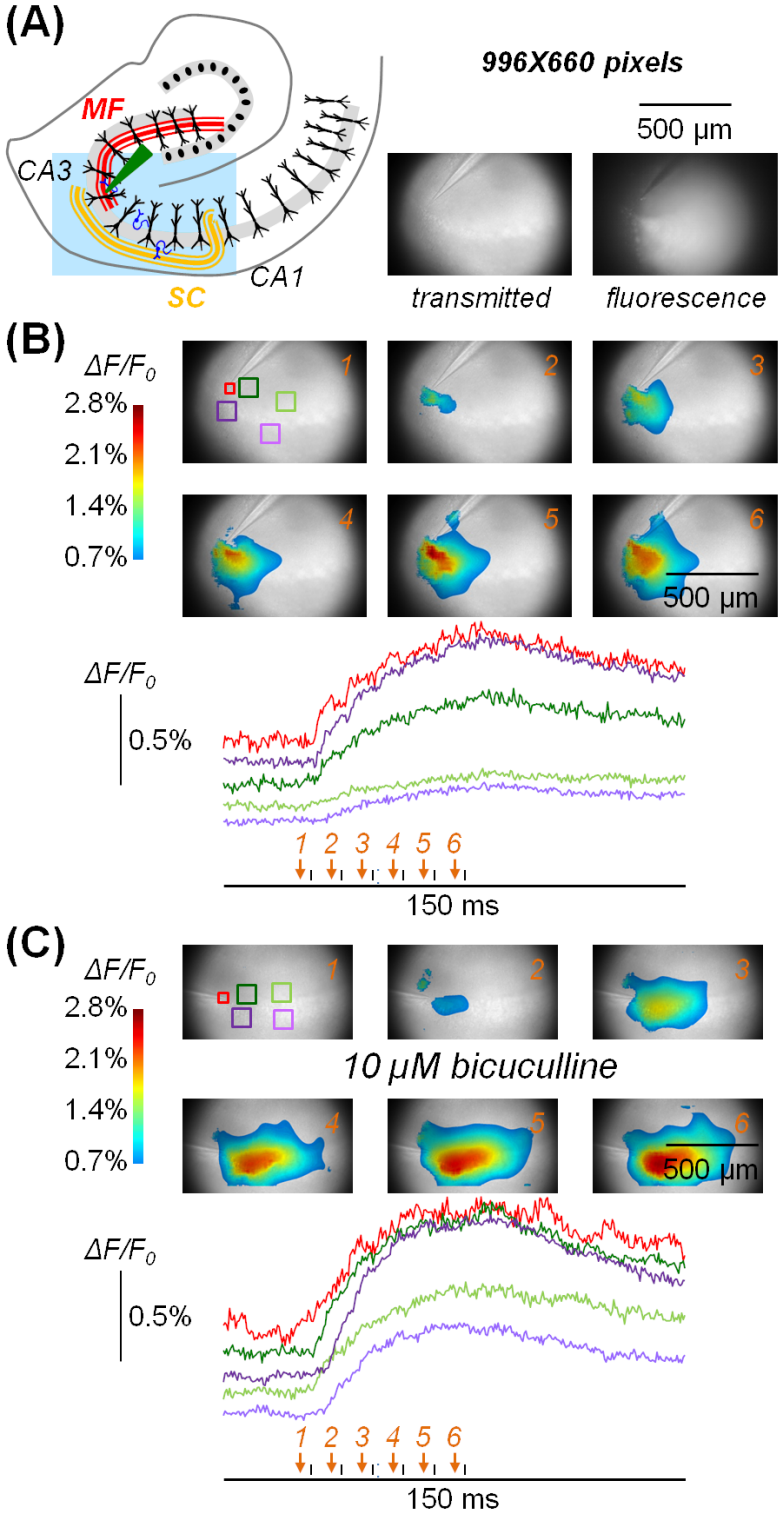
Imaging Ca^2+^ fluorescence at 2 kHz at 8‐bit depth from large fields. (A) Left, schematics of a hippocampal section with different regions indicated; the light blue rectangle represents the area of recording (996 × 660 pixels) with the position of a stimulating electrode (green triangle) on the MF pathway. Right, recording area under transmitted light and fluorescence. (B) Top, Δ*F*/*F*_0_ signal associated with 6 pulses at 10 ms interval illustrated using a coloured scale and superimposed to the transmitted light image; the six frames were captured at the times indicated by the arrows on the bottom; five colour‐outlined ROIs are indicated over frame *1*. Bottom, Δ*F*/*F*_0_ signal in the ROIs reported on the top. This experiment was performed in regular extracellular solution. Bottom, Δ*F*/*F*_0_ signal in the five colour‐outlined ROIs indicated on frame *1* on the top. This experiment was performed in control extracellular solution. (C) Top, in another slice during perfusion with extracellular solution containing 10 μM bicuculline, Δ*F*/*F*_0_ signal associated with the same stimulating protocol illustrated using a coloured scale, with the six frames captured at the times indicated by the arrows on the bottom.

According to camera specifications, it is possible to record 8‐bit frames at 2 kHz from nearly the entire field of the objective if the image is demagnified by 0.25× (see Figure 1). Figure 3A (left) shows a schematic drawing of a hippocampal section with a light‐blue rectangle positioned on the area visualised by the camera with the 25× objective. This area of 996 × 660 pixels with ~1 μm per pixel could be sampled at 8‐bit depth at 2000 frames/s. The two images of the hippocampal slice in Figure 3A (right), including parts of the CA3 and of the CA1 regions, have been taken under transmitted light (at 16‐bit depth) and under fluorescence (at 8‐bit depth), after staining the slice with the Ca^2+^ indicator Fluo‐4 AM which has been extensively used in brain slice experiments [25, 26, 27, 28]. Notably, the result of the staining protocol is a relative homogeneous fluorescence over the entire slice surface, allowing the possibility of collecting between 100 and 200 photons in most pixels. In this proof‐of‐principle experiment, we positioned a stimulating electrode (green triangle) at the end of the *stratum lucidum* in the last part of the MF pathway and we applied a stimulation protocol consisting of six current pulses delivered at 100 Hz. To initially analyse the spatial and temporal resolution of the Ca^2+^ transient elicited by the stimulation, we applied 41 × 41 offline binning and further filtered each frame of the sequence with an 80‐pixel two‐dimensional Wiener algorithm (“Wiener2” Matlab function, [29]) applied three times. Figure 3B (top) shows a sequence of frames before and during the stimulation protocol where Δ*F*/*F*_0_ (obtained from the average of three trials) are illustrated using a colour scale superimposed on the transmitted light image. Specifically, Δ*F*/*F*_0_ values above 2.8% are depicted with dark red tone whereas values below 0.7% (close to the noise level) are not displayed. The signal started near the electrode after the first stimulating pulse (frame 2, see timing on the bottom) and spread toward the CA1 region during the pulse train (frames 3–6) up to ~250 μm from the stimulating electrode. The full evolution of the Δ*F*/*F*_0_ signal at reduced time scale before, during and after the pulse train over an interval of 150 ms can be appreciated in Video S1 (available in the Supporting Information). To analyse the time‐course of the signal and its SNR, we averaged fluorescence over ROIs of 50 × 50 pixels or 100 × 100 pixels from raw data (equivalent to offline binning). Figure 3B (bottom) shows Δ*F*/*F*_0_ signals from the five colour‐outlined ROIs illustrated above, where *γ* was > 100 photons in each pixel. In the red ROI, centred over the tip of the stimulating electrode, and in the other ROIs positioned at different sites of the slice, the photon noise corresponded to a fractional change of fluorescence < 0.2% and Δ*F*/*F*_0_ signals of 1% or larger could be measured with outstanding SNR. In another slice experiment, we blocked feedforward and feedback synaptic inhibition that prevents hyperexcitable states [30] by perfusing the slice with extracellular solution containing 10 μM of the GABA_A_ receptors antagonist bicuculline. Under this condition mimicking epilepsy [31, 32, 33], we performed the same experiment described above. In this case, we observed Δ*F*/*F*_0_ transients > 1% beyond 500 μm from the stimulating electrode, delayed by 3–4 frames (Figure 3*C*, the evolution of the Δ*F*/*F*_0_ signal in this example can be appreciated in Video S2, available in the Supporting Information). In summary, we demonstrated that Ca^2+^ signals corresponding to Δ*F*/*F*_0_ < 1% can be resolved at 2 kHz over the entire field of the objective by performing offline binning.

### 3.3 Imaging Dendritic Ca^2+^ Transients Associated Back‐Propagation of Action Potentials at 10 kHz

**FIGURE 4:**
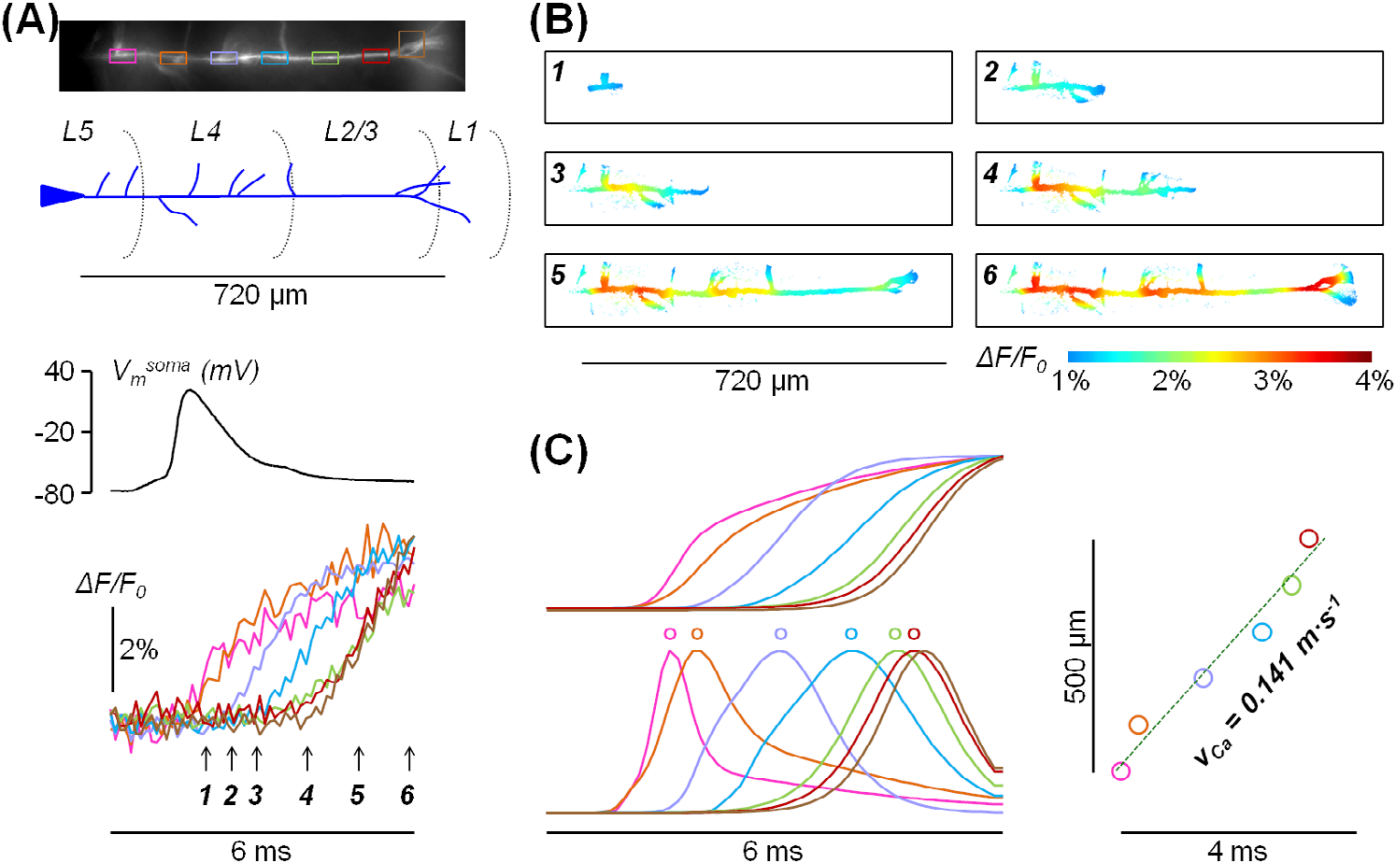
Imaging Ca^2+^ fluorescence at 10 kHz at 8‐bit depth. (A) Top, fluorescence image of the apical dendrite of a L5 pyramidal neuron with positions of L5, layer‐4 (L4), layers‐2/3 (L2/3) and L1 indicated below; the frame, composed of 800 × 140 pixels, is the size allowing imaging at 8‐bit depth. Bottom, somatic AP and associated Ca^2+^ transients from the rectangular coloured ROIs depicted on the image on the top. (B) Δ*F*/*F*_0_ signal illustrated using a coloured scale corresponding to the six frames captured at the times indicated by the arrows in panel a. (C) Left, fits of the Ca^2+^ transients in panel a using a 3‐sigmoid function and associated Ca^2+^ currents calculated as time derivatives; the timings of the current peaks are indicated by circles. Right, current peaks against time with the linear fit (dotted line) showing the velocity of Ca^2+^ propagation (*V*_Ca_).

In layer‐5 (L5) pyramidal neurons, APs actively propagate back into the dendritic tree [34, 35, 36, 37, 38], causing transient elevations of intracellular Ca^2+^ concentration [39, 40, 41]. Several studies have shown that all high‐voltage activated voltage‐gated Ca^2+^ channels (VGCCs) contribute to this Ca^2+^ transient [21, 39, 42] and these channels can mediate regenerative potentials in the case of AP bursts at high frequencies [43]. In this system, we recently performed a study of Ca^2+^ transients and currents associated with the back‐propagating AP [21], using the low affinity indicator OG5N at 20 kHz from 26 × 4 pixels frames, using a CCD camera. In that study, however, we focussed only at sites of the apical dendrite located at ~100 μm from the soma. With the camera described in this report, we were able to record fluorescence at 10 kHz and 8‐bit depth over the much larger frame size of 800 × 140 pixels. As shown in Figure 4*A* (top), when using the 25× objective and the 0.25× demagnifier, the frame (~832 × 146 μm^2^) covered the entire apical dendrite from L5 to layer‐1 (L1). We evoked an AP by somatic current injection via the patch pipette (Figure 4A, bottom) and averaged Ca^2+^ fluorescence over several 50 × 25 pixels ROIs, at different distances from the soma, and from a 50 × 50 pixels ROI positioned on the tuft (Figure 4A, bottom). Thus, from each ROI, we collected > 100 000 photons and by averaging nine individual trials, we could reduce the fluorescence fluctuations due to the photon noise to less than 0.4% (Figure 4A, bottom), achieving the SNR attained previously with a CCD camera [21]. Traces in Figure 4A (bottom) show the progressive delay in the rise of the Ca^2+^ transient as the AP back‐propagates towards the dendritic tuft. To illustrate the spatial and temporal profile of the Ca^2+^ transient associated with AP back‐propagation, we applied to raw data an 8 × 8 pixels median filter and a mask to eliminate dark pixels. Figure 4B shows six frames illustrated using a colour scale where Δ*F*/*F*_0_ values above 4% are depicted with the dark red tone whereas values below 1% are not shown. Each frame corresponds to a time during and after the recorded somatic AP (arrows in Figure 4A, bottom) and the full evolution of the Δ*F*/*F*_0_ signal over an interval of 6 ms can be appreciated in Video S3 (available in the Supporting Information). As it was done in previous studies on hippocampal CA1 pyramidal neuron (see sect. 3.3 in [20]), we fitted the Δ*F*/*F*_0_ signals at each of the seven positions along the apical dendrite with a 3‐sigmoid function (Figure 4C, left‐top). We have previously demonstrated that when the Ca^2+^ Δ*F*/*F*_0_ signal reaches a steady‐state level, its time derivative tracks the kinetics of the Ca^2+^ current [44]. Thus, we calculated the time derivatives in each position in order to extract the kinetics of Ca^2+^ currents at each position (Figure 4 *C*, left‐bottom). We set to *t* = 0 the time of the peak of the most proximal Ca^2+^ current and plotted the distance from this site against the time of the Ca^2+^ current peak for all the seven positions (Figure 4 *C*, right). Then, by fitting the first six points linearly, excluding the last on the tuft, we extracted the mean velocity of Ca^2+^ propagation (*V*_Ca_) which was in this experiment 0.141 m/s. Notably, a speed of ~0.15 m/s was also found in another cell tested. These values can be compared with the mean velocity of AP propagation which is 0.5 m/s on average [35] and varies from ~1 m/s near the soma to ~0.3 m/s at 500 μm from the soma [45]. Although Ca^2+^ propagation is faster between the first two points near the soma (Figure 4C), *V*_Ca_ was everywhere considerably slower with respect to the AP velocity. This is due to the fact that *V*_Ca_ provides a measurement of diverse VGCC activation that is delayed with respect to the AP peak [21]. The speed of VGCC depends on *V*_m_ reached by the AP at each site, and since the AP amplitude attenuates with distance from the soma, the Ca^2+^ current progressively slows down as we previously demonstrated in hippocampal CA1 pyramidal neurons [20]. In summary, the ability to measure Ca^2+^ currents along the entire apical dendrite of a L5 pyramidal neuron (> 500 μm) represents an important step‐forward in the study of VGCCs associated with back‐propagating APs.

### 3.4 Imaging the Action Potential Generation in the Axon Initial Segment at 40 kHz

**FIGURE 5:**
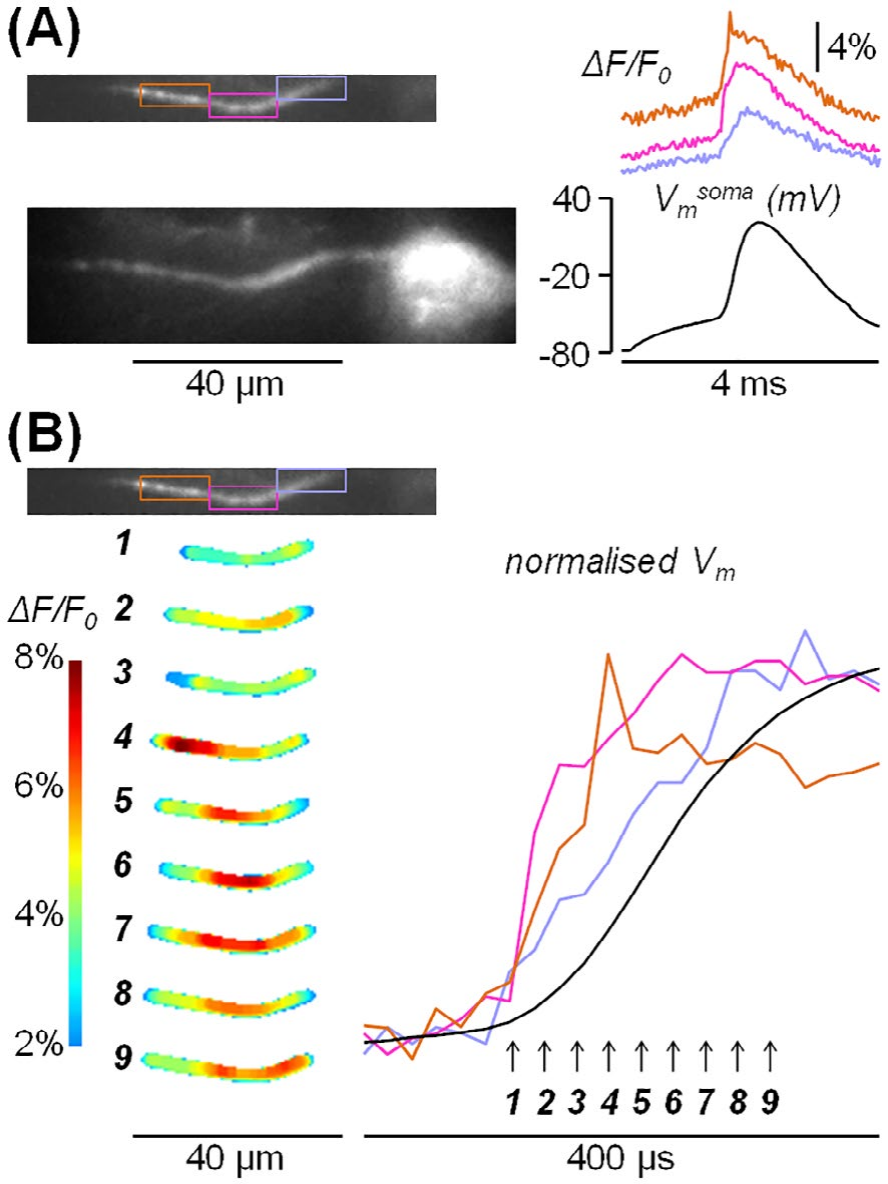
Imaging *V*_m_ fluorescence at 40 kHz at 8‐bit depth. (A) Left, fluorescence images of the AIS of a L5 pyramidal neuron: Top, frame composed of 180 × 21 pixels allowing imaging at 40 kHz at 8‐bit depth; bottom, larger frame including the same AIS and the soma. Right, somatic AP and associated *V*_m_ transients from the rectangular coloured ROIs depicted in the image on the left. (B) Left, Δ*F*/*F*_0_ signal illustrated using a coloured scale corresponding to the nine frames captured at the times indicated by the arrows on the right. Right, normalised *V*_m_ transients in the same ROIs in panel a over a temporal scale of 400 μs.

The last proof‐of‐principle experiment was designed to address the question of whether the present imaging mode is capable of resolving physiological signals at unprecedented temporal resolution. The signal that we selected for this test was the generating AP in the axon initial segment (AIS) [46]. In the AIS of L5 pyramidal neurons, the AP was measured using *V*_m_ imaging first by Palmer and Stuart [47], and then by others [17, 18, 48, 49, 50, 51]. All these measurements were performed at the temporal resolution of 50–100 μs and imaging at 20 kHz could be achieved from rectangles of 26 × 4 pixels only, using a fast CCD camera. Yet, the AP shape in the AIS, which is sharpened by voltage‐gated K^+^ channels [52] and by high Na^+^ channel density [53], requires higher acquisition rates to be fully reconstructed [49]. After loading a L5 pyramidal neuron with the VSD JPW1114, we imaged *V*_m_ fluorescence from the AIS at 40 kHz, with 8‐bit depth, from a rectangle of 180 × 21 pixels (Figure 5A, left), corresponding to ~78 × 9 μm^2^ when using the 60× objective. We evoked an AP by somatic current injection via the patch pipette (Figure 5A, right) and averaged *V*_m_ fluorescence over three 30 × 10 pixels rectangles, corresponding to three different positions of the AIS, collecting > 25 000 photons from each rectangle. Then, by averaging nine individual trials, we reduced fluorescence fluctuations due to photon noise to less than 1%, achieving the same SNR attained in previous measurements at lower acquisition rates [17, 18]. To analyse the spatial and temporal profile of the AP rise, we applied to raw data an 8 × 8 pixels median filter and a mask to eliminate dark pixels. Figure 5B (left) shows the sequence of nine frames during the AP rise illustrated using a colour scale where Δ*F*/*F*_0_ values above 8% are depicted with the dark red tone whereas values below 2% are not shown. The full evolution of the Δ*F*/*F*_0_ signal during the AP rise over 400 μs can be appreciated in Video S4 (available in the Supporting Information). In this experiment, the AP reaches its peak at frame 4 (Figure 5B, left) in the distal part of the AIS (~35 μm from the soma) and at frame 6 in the medial part of the AIS (~20 μm from the soma). Moreover, the normalised Δ*F*/*F*_0_ values reported in Figure 5B (right) indicate that the slope of the AP rise is steeper in the distal and medial parts of the AIS with respect to the proximal part (~10 μm from the soma). Thus, we demonstrated that the *V*_m_ transient associated with the generation of the AP in the AIS can be imaged at 8‐bit depth and that imaging at 25 μs temporal resolution allows discriminating the slope of the AP rise at different axonal sites.

## 4 Discussion

In this technical report, we demonstrate that fast fluorescence transients, from Ca^2+^ or *V*_m_ indicators, can be resolved at 8‐bit depth with outstanding SNR, even when Δ*F*/*F*_0_ is below 1%. The strategy consists in assigning to each pixel the summation of photoelectrons of surrounding pixels (offline binning), this way increasing both the digital resolution and the number of photoelectrons. As a reference, the noise of Ca^2+^ Δ*F*/*F*_0_ in the 50 × 25 pixels ROIs reported in Figure 4A was ~0.4% in the averages of nine trials, identical to the photon noise associated with previous measurements of the same signal, from ROIs of the same size, using a CCD camera [21]. The challenge was to assess this approach with respect to the dramatic gain in acquisition speed that can be achieved by sampling light intensity signals at 8‐bit depth, in particular with frames of tens or hundreds thousands pixels. The camera's sensor, comprising over 10 M pixels, can observe the entire field of a microscope objective without magnification, but demagnification allows enlarging the area of the specimen that can be sampled at a given speed (Figure 1). In the proof‐of‐principles examples, after 0.25× demagnification, the pixel covered an area of either ~1 μm or ~0.5 μm depending on the utilised objective. A 5 × 5 offline binning operation, necessary to increase the effective digital depth above 12‐bit, worsens the spatial resolution to ~5 μm maximum still preserving the ability to discriminate signals from relatively small cellular compartments or single neurons in networks. The sizes of the frames obtained at 2 kHz or 10 kHz with the present device should be compared with those achieved in the past either with CCD or with CMOS devices (see Introduction) when acquiring signals at 14‐bit depth. With the limited frame sizes constrained by the higher bit depth, the choice between imaging from restricted portions of the specimen with high pixel resolution, or from larger portions with low pixel resolution had to be done. Here, we achieved imaging at 2 kHz with a frame size of 990 × 660 pixels (Figure 3B) or at 10 kHz with a frame size of 800 × 140 pixels (Figure 4A), in both cases observing a field of ~720 μm with a pixel resolution of ~1 μm. Since summating digital numbers from neighbouring pixels with comparable intensity values is necessary to resolve small fluorescence changes from 8‐bit data, the present method has still critical limitations in several applications. First, it cannot resolve a signal from small structures such as dendritic spines as it was achieved using camera imaging at higher bit depths [54]. Second, it cannot report activity from large fields if emitted fluorescence is not sufficiently uniform, since in this case either the dimmest areas would be effectively sampled at lower bit depth or, alternatively, the brightest areas would saturate the EWC. This limitation, however, can be overcome by spatially modulating the excitation light intensity, for instance using holography [55, 56]. Finally, in the device used here, the 8‐bit acquisition mode functions as a photon counter with a digital unit corresponding to a photoelectron. This mode allows detecting low light levels from small areas during short time windows, but limits the amount of photons that can be detected and the size of signal that can be discriminated. Specifically, the 8‐bit acquisition mode in this device cannot resolve signals of Δ*F*/*F*_0_ ~ 0.1%, which is the need, for instance, of population *V*_m_ imaging in vivo [57]. This limitation can be potentially overcome by future cameras allowing for higher EWC in the 8‐bit acquisition mode, that is, with a digital unit corresponding to tens of photoelectrons. In summary, within present limitations, we demonstrated here that imaging at 8‐bit depth allows combining high spatial and high temporal resolution necessary to explore physiological signals from multiple sites, over large fields, including single cells and neuronal networks.

## Author Contributions

M.C. designed the research, F.A., Ö.Y.İ., and M.C. performed the experiments; M.C. performed data analysis; P.M. designed and built part of the set up; M.C. wrote the paper. All authors have given approval to the final version of the manuscript.

## Ethics Statement

Experiments in brain slices were performed at the Laboratory of Interdisciplinary Physics in Grenoble in accordance with European Directives 2010/63/UE on the care, welfare and treatment of animals. Procedures were reviewed by the ethics committee affiliated to the animal facility of the university (E3842110001).

## Conflicts of Interest

The authors declare no conflicts of interest.

## Supporting information


**Video S1.** Colour scale movies report the Ca^2+^ fractional change of fluorescence (∆*F*/*F*_0_) associated with MF electrical stimulation in hippocampal slices. The colours scale span from 0.7% to 2.8%. The experiment of Supplementary Movie 1 was performed under regular extracellular solution. The experiment of Supplementary Movie 2 was performed during perfusion with extracellular solution containing 10 μM bicuculline. The interval between two consecutive frames in the movie is 2 ms and each frame is the average of four frames captures at 2 kHz.

**Video S2.** Colour scale movies report the Ca^2+^ fractional change of fluorescence (∆*F*/*F*_0_) associated with MF electrical stimulation in hippocampal slices. The colours scale span from 0.7% to 2.8%. The experiment of Supplementary Movie 1 was performed under regular extracellular solution. The experiment of Supplementary Movie 2 was performed during perfusion with extracellular solution containing 10 μM bicuculline. The interval between two consecutive frames in the movie is 2 ms and each frame is the average of four frames captures at 2 kHz.

**Video S3.** Colour scale movie reports the Ca^2+^ fractional change of fluorescence (∆*F*/*F*_0_) in the apical dendrite of a L5 pyramidal neuron associated with a back‐propagating action potential elicited in the soma and reported on the left. The colours scale span from 1% to 4%. The interval between two consecutive frames is 100 μs.

**Video S4.** Colour scale movie reports the *V*_m_ fractional change of fluorescence (∆*F*/*F*_0_) in the axon initial segment of a L5 pyramidal neuron associated with the generation of an action potential. The somatic action potential is reported on the right. The colours scale span from 2% to 8%. The interval between two consecutive frames is 25 μs.

## Data Availability

The data that support the findings of this study are openly available in Zenodo at https://zenodo.org/records/13365448, reference number DOI: https://doi.org/10.5281/zenodo.13365447.
